# Predictive Inference Alterations in Psychosis Proneness Show Task‐Dependent Signatures

**DOI:** 10.1111/nyas.70347

**Published:** 2026-07-24

**Authors:** Luca Tarasi, Vincenzo Romei

**Affiliations:** ^1^ Centro Studi e Ricerche in Neuroscienze Cognitive, Dipartimento di Psicologia “Renzo Canestrari”, Campus di Cesena Alma Mater Studiorum – Università di Bologna Bologna Italy; ^2^ Universidad Antonio de Nebrija Madrid Spain

**Keywords:** history biases, perceptual decision‐making, predictive coding, psychosis‐like experiences, schizotypy, serial dependence

## Abstract

Perception relies on the dynamic integration of sensory inputs with prior expectations shaped by recent experience. Disruptions in this inferential process are thought to underlie psychosis, yet whether similar alterations occur in subclinical psychosis‐like experiences (PLEs) and how they manifest across perceptual contexts remain unclear. Here, we tested two independent samples of healthy adults: 80 performed a visual detection task and 62 a motion discrimination task. Mixed‐effects models revealed that higher PLE scores predicted reduced weighting of sensory evidence and greater reliance on probabilistic cues across tasks. Complementary signal detection analyses confirmed that higher PLE scores were associated with reduced perceptual sensitivity, alongside increased cue‐dependent response bias shifts. To capture how recent experience shapes perception, we quantified history biases: the influence of the previous stimulus or choice on current decisions. These biases diverged across contexts: in the detection task, the repulsive bias from the previous stimulus was attenuated in high‐PLE individuals, whereas in the discrimination task, they exhibited a repulsive bias away from previous choices. These task‐dependent signatures reveal that psychosis‐like traits differentially alter the use of past information, pointing to a broader inferential vulnerability along the psychosis continuum.

## Graphical abstract

How do psychosis‐like traits reshape the balance between what we sense and what we expect? Across two perceptual decision tasks, we show that higher psychosis proneness reduces reliance on sensory evidence while amplifying the influence of prior expectations. Critically, the way past experiences bias current decisions depends on task demands—revealing not a uniform deficit, but a task‐dependent reconfiguration of predictive inference along the psychosis continuum.

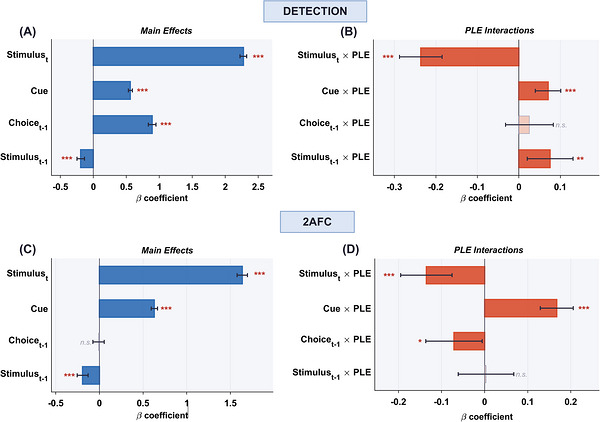

## 1 Introduction

Perception is not a passive readout of sensory input, but an active inferential process whereby the brain integrates sensory evidence with prior knowledge to generate probabilistic estimates of the external world. This view, formalized in predictive processing theories, holds that perceptual experience arises from the interplay between bottom‐up signals and top‐down predictions, shaped by the minimization of prediction error [1, 2]. Crucially, when the balance between sensory evidence and prior expectations becomes dysregulated, perception itself can become distorted—leading individuals to perceive expected stimuli in the absence of reliable evidence, or to misinterpret ambiguous inputs in line with internal predictions [3, 4]. Growing evidence suggests that such computational imbalances lie at the core of psychosis‐spectrum phenomena, spanning from attenuated symptoms to full‐blown schizophrenia [5, 6, 7]. For example, hallucinations have been proposed to reflect an overreliance on prior beliefs, which dominate perception even in the absence of reliable sensory input [3, 8], reflecting a shift in the relative weighting of prior expectations versus sensory evidence under uncertainty. Delusions, in turn, may emerge when low‐level sensory priors are imprecise, allowing spurious signals to gain undue significance. This uncertainty may trigger a compensatory shift at higher hierarchical levels, where cognitive priors become more influential relative to unreliable sensory signals, constraining interpretation in rigid, belief‐consistent ways [9, 10]. These aberrations in perceptual inference would reflect a fundamental miscalibration in the weighting of internal predictions versus external input.

Importantly, similar computational deviations have been documented in the general population. Psychosis‐like experiences (PLEs)—unusual perceptual or belief‐related phenomena occurring in the absence of a clinical disorder—are increasingly recognized as meaningful subclinical expressions of psychosis vulnerability [11, 12, 13]. Recent findings indicate that psychosis proneness is associated with altered weighting of sensory evidence and prior information, with the direction and magnitude of these effects varying across paradigms and across prior sources [14, 15, 16, 17]. These results support a dimensional view of psychosis, in which disruptions to predictive coding mechanisms unfold along a continuum and may be detectable well before the emergence of clinical symptoms.

In addition to the integration of priors and sensory evidence, perceptual inference also relies critically on recent experience. Humans display robust sequential biases, whereby current choices are influenced by preceding stimuli and responses [18, 19, 20, 21]. These serial dependencies are increasingly viewed as expressions of predictive inference, reflecting the brain's tendency to exploit temporal regularities to optimize perception under uncertainty. Although sequential biases typically enhance perceptual stability, they appear disrupted in psychosis, with altered history effects reported in both clinical and high‐risk populations [22, 23, 24], although not all findings are consistent [25]. A key open question is whether these alterations reflect a stable, trait‐like vulnerability marker, or rather a task‐sensitive maladaptation—whereby perceptual history is used inconsistently depending on context demands.

To date, few studies have systematically investigated how psychosis proneness shapes the integration of sensory evidence, prior expectations, and choice history across tasks that differ in their hierarchical and computational demands. Critically, while altered serial dependence has often been interpreted as a general marker of impaired predictive processing in psychosis, such accounts may overlook the possibility that these biases also reflect a maladaptive sensitivity to task structure—leading to unstable or context‐inappropriate inference strategies depending on the hierarchical nature of the decision.

Here, we directly address this gap by combining two well‐characterized paradigms that probe distinct forms of perceptual uncertainty. In the first, a visual detection task, participants judged the presence or absence of near‐threshold stimuli. In the second, a motion discrimination task, participants resolved directional uncertainty using dynamic dot patterns that require continuous evidence accumulation [26]. Although both tasks were titrated to yield comparable accuracy, they differ fundamentally in their computational demands and the level of processing they engage. Specifically, the detection task places greater demands on early sensory encoding, where perceptual history may influence subsequent trials via low‐level adaptation mechanisms. In contrast, the discrimination task draws more heavily on internal decision dynamics, making it more sensitive to how previous choices influence current behavior. This distinction provides a framework to test whether psychosis‐like traits differentially affect the integration of past information across the sensory–decisional axis—disrupting stimulus‐driven biases when adaptation is key, and altering choice‐related biases in tasks that rely more heavily on decision‐level processing.

Using generalized linear mixed models and signal detection theory (SDT) modeling, we quantified the influence of current sensory input, prior cues, previous stimuli, and previous choices on perceptual decisions and, critically, how these factors interact with individual differences in psychosis proneness.

Our results indicate that elevated PLEs are associated with reduced stimulus weighting in choice and stronger use of explicit cue‐induced expectations across tasks, consistent with predictive coding accounts. SDT analyses indicate that this pattern is consistent with both a general reduction in perceptual sensitivity and amplified cue‐dependent criterion shifts, suggesting that sensory and decisional mechanisms jointly contribute to altered evidence weighting in psychosis‐prone individuals. Crucially, sequential biases revealed task‐dependent effects: in the detection task, which relies more on sensory mechanisms, high‐PLE individuals showed an attenuation of the typical repulsive bias from the previous stimulus (but not choice), whereas in the discrimination task, which relies more on decisional mechanisms, they exhibited a repulsive bias relative to their previous choice (but not stimulus). These results indicate that psychosis‐prone individuals do not exhibit a uniform reduction in serial dependence, but rather task‐dependent alterations in the integration of past information across different inferential contexts. Such differential use of sensory and decisional history may reflect early alterations in the hierarchical architecture supporting predictive inference.

## 2 Materials and Methods

We conducted two behavioral experiments to examine how prior expectations and sequential dependencies influence perceptual decisions as a function of psychosis proneness. A total of 142 healthy adults participated across two independent samples (Experiment 1: *n* = 80; Experiment 2: *n* = 62); no participant took part in both experiments. Sample sizes were determined based on comparable prior behavioral studies investigating perceptual decision‐making/individual differences in psychosis proneness [21, 22, 27]. No participants were excluded from the analyses. Participants were healthy, White young adults (see Table 1 for age, sex, education, and Schizotypal Personality Questionnaire [SPQ]‐derived PLE scores). Testing took place in a lab, in a quiet room under controlled lighting conditions. Inclusion criteria were age range (18−35), normal or corrected‐to‐normal vision, no self‐reported history of neurological or psychiatric disorders, and no current psychoactive medication. Participants were recruited via university participant pools and via flyers. All participants signed a written informed consent prior to taking part in the study, which was approved by the Bioethics Committee of the University of Bologna (protocol no. 201723, approval date: August 26, 2021), and conducted in accordance with the Declaration of Helsinki.

**TABLE 1:** Demographic characteristics and psychosis‐like experiences across experimental tasks.

	Detection task	Random dot motion discrimination task
Sex (F/M)	43/37	37/25
Age (years)	23.79 ± 2.79	23.68 ± 3.13
Education (years)	18.48 ± 2.22	18.52 ± 2.80
PLEs (mean ± SD)	6.38 ± 5.33	2.92 ± 3.44
PLEs (range)	0−20	0−16

*Note*: Demographic information and psychosis‐like experience (PLE) scores for participants in the visual detection task (*N* = 80) and the visual random dot motion discrimination task (*N* = 62). Values are reported as mean ± standard deviation (SD) for continuous variables and as counts for categorical variables (sex). PLEs were computed by summing scores from three subscales of the Schizotypal Personality Questionnaire (SPQ): Magical Thinking, Ideas of Reference, and Unusual Perceptual Experiences. PLE, psychosis‐like experience.

### 2.1 Experiment 1: Detection Task

**FIGURE 1:**
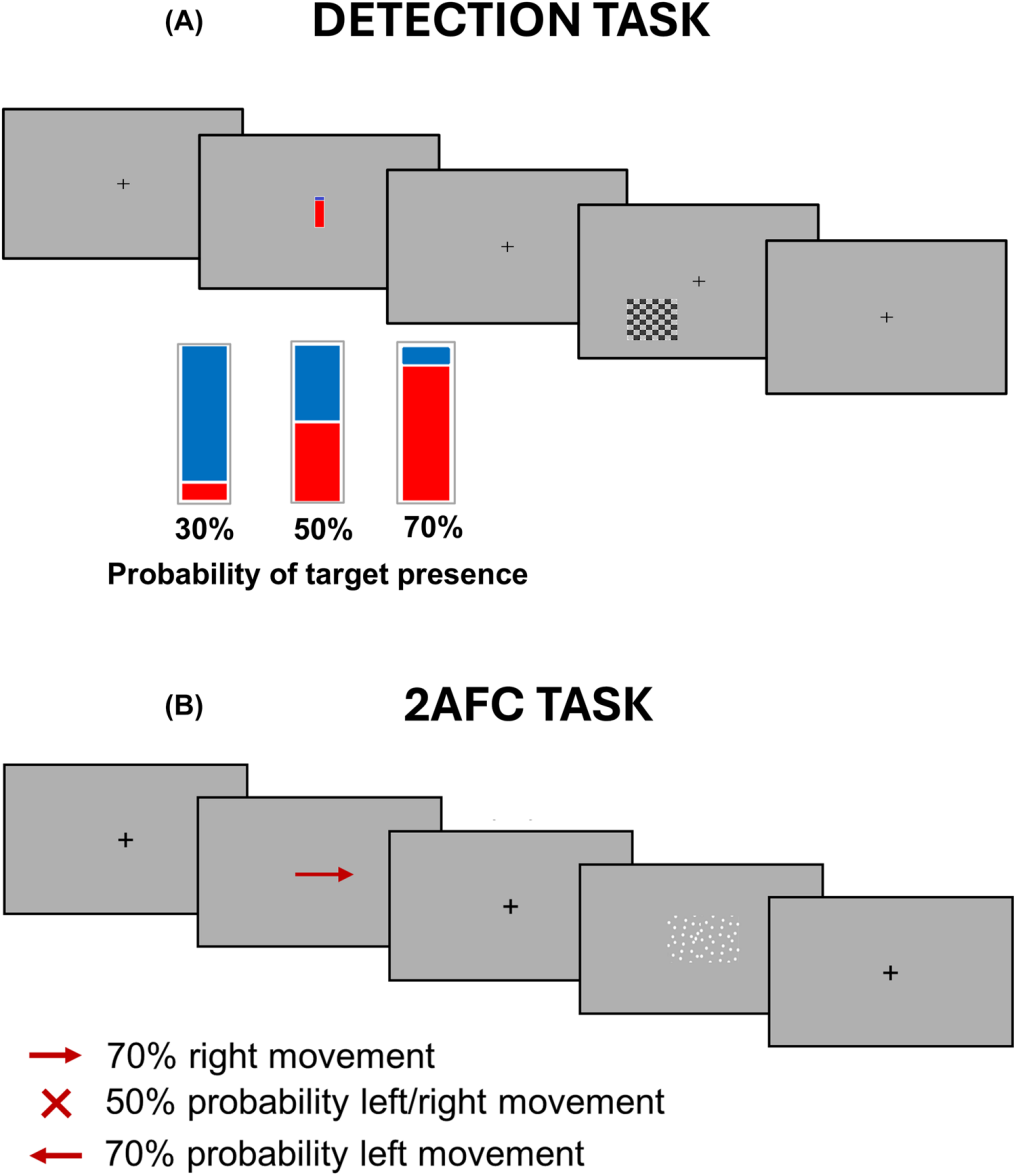
Probabilistic detection task and 2AFC random dot motion task. (A) Each trial began with a central cue—a vertically split bar (red bottom, blue top)—displayed for 1 s. The bar fill level indicated the probability of target presence: high (70%), low (30%), or neutral (50%). After a variable delay (1.2–1.5 s), a checkerboard with or without gray circles appeared for 0.06 s, followed by a black screen (1.9–2.4 s). (B) Each trial began with a probabilistic cue indicating the likelihood of leftward or rightward motion. After a 1 s delay, a centrally presented dot motion stimulus appeared for 0.4 s. Cues were either informative (arrows indicating 70% probability) or uninformative (a cross indicating 50%). In both tasks, cue validity matched actual target probability, and participants were informed accordingly. Stimulus contrast (checkerboard) and motion coherence (dots) were titrated to achieve ∼70% accuracy. Abbreviation: 2AFC, two‐alternative forced choice.

Participants performed a probabilistic detection task designed to assess how perceptual decisions are shaped by varying levels of sensory expectation [28, 29, 30, 31] (Figure 1A). Stimuli were presented on an 18‐inch CRT display at approximately 57 cm viewing distance in a dimly lit room, using MATLAB (R2016b) and the Psychophysics Toolbox. Visual stimuli consisted of 8 × 8 black‐and‐white checkerboards (4 cm height/width), had a spatial frequency of 5.16 cycles/degree, and they were presented only in the lower‐left part of the screen at 4.1°/3.7° eccentricity (horizontal/vertical). Target stimuli consisted of iso‐luminant gray circles (diameter = 0.5 cm) embedded within the checkerboard cells. The RGB contrast of the circles could range from 15/240 (minimum displayable contrast on white/black checkerboard squares, respectively) to 75/180 (maximum contrast). Catch trials consisted of identical checkerboard patterns without the embedded gray circles (i.e., target‐absent trials with RGB contrasts of 0/255). The task comprised two phases. In the initial phase, an adaptive psychophysical procedure was used to determine each participant's contrast threshold for detecting gray circular targets. This titration yielded ∼70% accuracy across a balanced mix of target‐present and catch trials [32, 33]. In the main experiment, each trial began with a visual probabilistic cue indicating the likelihood that a target would appear. When present, the target consisted of a gray circle embedded within a checkerboard background, displayed in the lower‐left quadrant of the screen for 60 ms. Each trial began with a cue presented for a fixed duration of 1 s, followed by a variable interval of 1.2–1.5 s, after which the checkerboard appeared either with or without the target. Participants were instructed to respond using their right hand, pressing the “K” key if they detected the target, or “M” if they perceived its absence. No time pressure was imposed. After each response, a black screen was presented for a randomly varied interval ranging between 1.9 and 2.4 s. The probabilistic cue took the form of a vertically split rectangle, shaded red on the bottom and blue on the top. The proportion of red visually encoded the likelihood of target occurrence. There were three cue conditions: high probability, low probability, and a neutral condition, which provided no predictive information. Importantly, the actual frequency of target appearances across conditions matched the probabilities conveyed by the cues (i.e., target‐present and target‐absent trial proportions varied as a function of the probabilistic cue), and participants were explicitly informed about this correspondence.

### 2.2 Experiment 2: Random Dot Motion Discrimination Task

Participants performed a motion discrimination task using dynamic random‐dot stimuli composed of 400 white dots displayed within a central square region [34] (Figure 1B). Stimuli moved at a fixed velocity of 4.5°/s and were presented on a monitor positioned 70 cm from the seated participants in a dim environment, utilizing MATLAB (v2016b) and Psychophysics Toolbox. The stimuli varied in coherence—the proportion of dots moving uniformly either to the left or right—across several difficulty levels (0%, 3%, 6%, 9%, 15%, 21%, 30%, and 60%). At 0% coherence, dots moved randomly, whereas at higher coherence levels, an increasing proportion moved consistently in one direction, thus manipulating task difficulty. An individualized coherence threshold, corresponding to 70% accuracy, was determined beforehand for each participant via a titration phase employing the constant stimuli method and fitting a psychometric function with the *psignifit* toolbox [35]. In each experimental trial, participants indicated the perceived dot motion direction (left or right) by pressing designated keys (F5 for leftward, F12 for rightward) using their index finger. Before the main task, participants completed a brief demonstration followed by a training phase to familiarize themselves with task requirements. The main experiment consisted of four blocks of 120 trials each, during which directional expectations were manipulated by probabilistic cues presented 1 s before stimulus onset. Cues were either informative (left‐ or right‐pointing arrows) or uninformative (a central cross). Informative cues indicated the more likely upcoming motion direction and were valid on 70% of trials (invalid on 30%), whereas the uninformative cue corresponded to a 50/50 probability. Each trial began with a fixation period lasting 3.5 s, followed by the probabilistic cue and the subsequent stimulus presented at the predetermined coherence level for 400 ms. Participants were explicitly informed of cue validity to ensure the correct interpretation of the probability information provided. In both the detection and random dot motion tasks, only individually calibrated at‐threshold stimuli were presented during the main experimental blocks.

### 2.3 Questionnaires

In both experiments, participants completed the SPQ [36], a widely used self‐report measure of schizotypal traits in nonclinical populations (Figure S1). The SPQ comprises subscales targeting cognitive‐perceptual, interpersonal, and disorganized features associated with the schizophrenia spectrum. For this study, we focused on a composite index of PLEs, computed by summing scores from three subscales: Magical Thinking (MT), Ideas of Reference (IoR), and Unusual Perceptual Experiences (UPE). Mean PLE scores were 6.38 ± 5.33 (SD) in Experiment 1 and 2.92 ± 3.44 in Experiment 2. These were selected for their strong theoretical and empirical relevance to anomalous beliefs and sensory distortions thought to index subclinical expressions of psychosis. This PLE index served as an individual‐differences variable to examine how variability in psychosis proneness modulates perceptual decision‐making and the integration of prior expectations. Suspiciousness, although sometimes included within broad positive‐schizotypy factors, was excluded because it indexes paranoid/interpersonal processes and shows consistent cross‐loadings with negative/interpersonal dimensions in factor‐analytic work, thereby reducing construct specificity for the cognitive–perceptual component targeted here [37, 38, 39].

### 2.4 Generalized Linear Mixed‐Effects Model Analysis

Behavioral responses were analyzed using a generalized linear mixed‐effects model (GLMM) with a binomial distribution and logit link function, implemented in MATLAB R2022b via the *fitglme* function. The dependent variable was the participant's binary choice (present vs. absent in Experiment 1; leftward vs. rightward in Experiment 2). The logistic model was defined as follows:

$$\begin{array}{ccc} \mathrm{Response} & ∼ & \mathrm{Stimulu}s_{t}+\mathrm{Stimulu}s_{t-1}+\mathrm{Choic}e_{t-1}+\mathrm{Cu}e_{t}+\mathrm{PLE}\\ & & +\;\mathrm{Stimulu}s_{t}*\mathrm{PLE}+\mathrm{Stimulu}s_{t-1}*\mathrm{PLE}+\mathrm{Choic}e_{t-1}\\ & & *\mathrm{PLE}+\mathrm{Cu}e_{t}*\mathrm{PLE}+\left(1|\mathrm{Subject}\right) \end{array}$$

History‐dependent effects were modeled by including lagged regressors for the previous stimulus and response (Stimulus_t−1_ and Choice_t−1_). The model included trial‐level predictors—current stimulus (Stimulus_t_), previous stimulus (Stimulus_t−1_), previous response (Choice_t−1_), and cue probability (Cue_t_)—as well as a subject‐level predictor indexing PLE. Interaction terms were defined a priori based on our hypotheses and following the approach of Eckert et al. [22]. Specifically, we tested interactions between PLE and (i) previous response (choice history); (ii) previous stimulus (stimulus history); (iii) cue probability (explicit probabilistic expectations); and (iv) current stimulus (sensory evidence). To preserve the temporal structure of the data, trials were sorted by subject and trial number; the first trial for each participant was excluded due to missing lagged information. A random intercept was included to account for interindividual variability. Including both Stimulus_t−1_ and Choice_t−1_ in the same model enabled us to estimate stimulus‐history and choice‐history effects while controlling for their shared variance, mitigating confounding arising from the intrinsic stimulus–response coupling. To assess robustness and aid interpretability, we additionally conducted an extreme groups tercile analysis contrasting participants in the lowest versus highest tercile of the composite PLE score (Figure S3). Finally, to formally test whether PLE‐related modulations of history biases differed between tasks, we additionally fitted a combined GLMM pooling trial‐level data from both experiments. The model included Task as a between‐subject factor and all Predictor × Task × PLE full factorial terms (i.e., all lower‐order main effects and interactions included):

$$\begin{array}{ccc} \mathrm{Response} & ∼ & \mathrm{Stimulu}s_{t}*\mathrm{Task}*\mathrm{PLE}+\mathrm{Cu}e_{t}*\mathrm{Task}*\mathrm{PLE}\\ & & +\mathrm{Choic}e_{t-1}*\mathrm{Task}*\mathrm{PLE}+\mathrm{Stimulu}s_{t-1}*\mathrm{Task}\\ & & *\mathrm{PLE}+\mathrm{Task}*\mathrm{PLE}+\left(1|\mathrm{Subject}\right) \end{array}$$

PLE scores were z‐scored within each sample prior to pooling, ensuring that the Task * PLE interactions reflect differences in how within‐sample PLE variation modulates predictors rather than between‐sample differences in mean PLE levels. Because both the direction and the task‐specificity of the predicted effects were specified a priori in the Introduction, we report one‐tailed *p*‐values for the two critical three‐way interactions (Task * PLE * Stimulus_t−1_ and Task * PLE * Choice_t−1_).

### 2.5 Exploratory Analyses on PLE Subdimensions

We performed exploratory analyses examining the three SPQ subscales that constituted our composite PLE measure: MT, IoR, and UPE. For each subscale, raw scores were z‐scored across participants using the same standardization approach adopted for the composite PLE regressor. First, within each task, we refit the same trial‐wise GLMM used in the main analysis, replacing the composite PLE regressor with each subscale regressor in turn. Thus, for each task, we estimated three models with identical fixed‐effect structure, including the critical interactions between the subscale and the task predictors indexing: (i) current sensory evidence; (ii) explicit probabilistic expectations; (iii) sensory history; and (iv) choice history. All models included a random intercept for participant. To formally test whether subscale effects differed across tasks, we additionally fit combined models pooling both tasks and including the task factor, thereby providing direct statistical tests of whether each subscale differentially modulated sensory, cue‐based, and history‐based influences across the two decision contexts.

### 2.6 Threshold Calibration and SDT Analyses

We quantified each participant's threshold estimated during the adaptive calibration procedure used to titrate task difficulty (detection task: contrast threshold; motion discrimination task: coherence threshold) and then tested whether thresholds covaried with PLE scores using linear regression models (Threshold ∼ PLE) separately for each experiment. Moreover, to provide a more direct SDT‐based characterization of whether psychosis proneness relates to perceptual sensitivity versus decision bias, we computed subject‐level SDT indices [40] separately for each cue level: sensitivity (d′) and criterion (c). In the detection task, d′ indexes participants’ ability to discriminate signal from noise, whereas in the two‐alternative forced choice (2AFC) task, it reflects their ability to extract the correct motion direction. Criterion provides a measure of response bias: in the detection task, lower values indicate a more liberal criterion (i.e., a greater tendency to report target presence), whereas in the 2AFC task, lower values indicate a leftward bias in direction reports. For each experiment, we then tested associations between these indices and psychosis proneness using linear mixed‐effects models with subject as a random intercept. Specifically, we fit models of the form:

$$d^{\prime }∼Cue+PLE+Cue*PLE+\left(1|Subject\right)$$

$$c∼Cue+PLE+Cue*PLE+\left(1|Subject\right)$$

## 3 Results

### 3.1 Behavioral Performance

Participants completed two perceptual decision‐making tasks designed to characterize the influence of bottom‐up sensory evidence, top‐down explicit expectations, and trial history, and to examine how these factors are modulated by PLEs. Performance was calibrated using individualized titration procedures, yielding near‐threshold accuracy in both experiments (Experiment 1: mean = 73.8%, SD = 8.3%; Experiment 2: mean = 71.2%, SD = 5.2%). The two samples (Table 1) did not differ in age (t_140_ = 0.22, *p* = 0.82) or sex distribution (χ^2^ = 0.50, *p* = 0.48); however, mean PLE scores were higher in the detection task compared with the discrimination task (t_140_ = 4.47, *p* < 0.01). To quantify the contribution of sensory, sequential, and expectation‐related factors, we fitted GLMMs to each dataset. Fixed effects included current stimulus, previous stimulus, previous response, and cue probability, as well as their interactions with PLE scores; random intercepts were specified for each participant. Trials were sorted by subject and trial number, with the first trial excluded to preserve lag structure. Variance inflation factors (VIFs) computed for the main‐effect predictors were all below 1.4 (Experiment 1: max VIF = 1.31; Experiment 2: max VIF = 1.22), indicating no multicollinearity issues.

### 3.2 Perceptual Decisions Are Shaped by Sensory Evidence, Prior Cues, and Sequential Context

**FIGURE 2:**
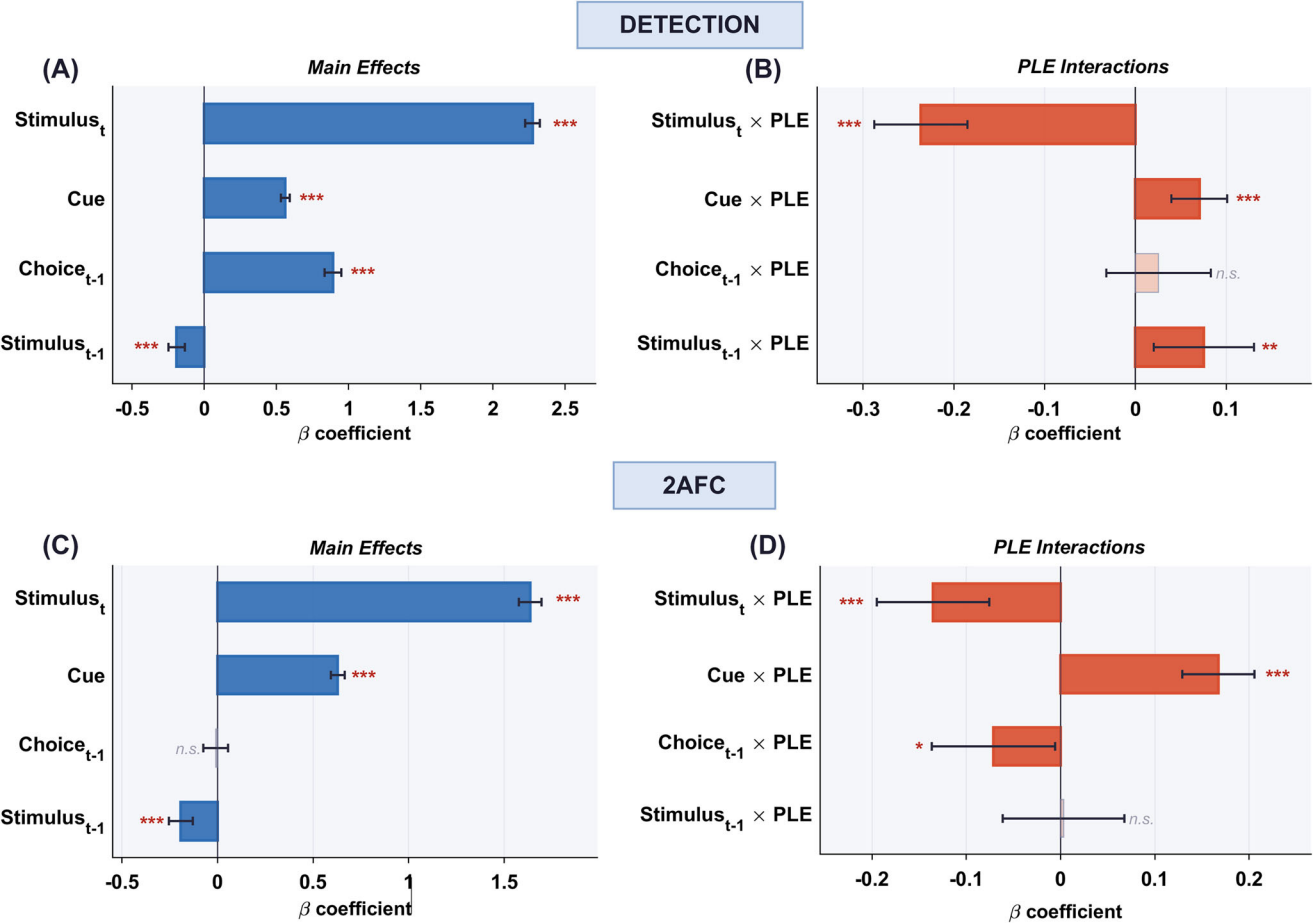
Beta coefficients of the logistic choice models for the detection task (A, B) and motion discrimination task (C, D). Left panels (A, C) display main‐effect coefficients; right panels (B, D) display PLE interaction coefficients. In both tasks, current sensory evidence (Stimulus_t_) and cue probability (Cue) positively predicted choice (Detection: β = 2.275, *p* < 0.001 and β = 0.561, *p* < 0.001; Discrimination: β = 1.636, *p* < 0.001 and β = 0.629, *p* < 0.001), while the previous stimulus (Stimulus_t−1_) exerted a repulsive effect (Detection: β = −0.191, *p* < 0.001; Discrimination: β = −0.192, *p* < 0.001). Previous choice (Choice_t−1_) showed a significant repetition bias in the detection task (β = 0.892, *p* < 0.001) but not in the discrimination task (β = −0.009, *p* = 0.787). PLE did not show a significant main effect in either task (Detection: β = 0.018, *p* = 0.801; Discrimination: β = 0.123, *p* = 0.058). Regarding PLE interactions, higher PLE scores were associated with task‐independent reduction of current sensory evidence weighting (Detection: β = −0.236, *p* < 0.001; Discrimination: β = −0.135, *p* < 0.001) and increased cue reliance (Detection: β = 0.071, *p* < 0.001; Discrimination: β = 0.167, *p* < 0.001). History‐related modulations were task‐dependent: in the detection task (B), higher PLE scores attenuated the repulsive influence of the previous stimulus (β = 0.075, *p* = 0.007) without affecting choice repetition (β = 0.026, *p* = 0.386); in the discrimination task (D), higher PLE scores shifted the previous‐choice effect toward alternation (β = −0.071, *p* = 0.033) without modulating previous‐stimulus effects (β = 0.003, *p* = 0.925). Bars indicate β estimates (log‐odds); error bars represent 95% confidence intervals. Saturated bars denote significant effects; desaturated bars denote nonsignificant effects. *p < 0.05; **p < 0.01; ***p < 0.001. Abbreviations: 2AFC, two‐alternative forced choice; PLE, psychosis‐like experiences.

Across both experiments (Figure 2), participants’ decisions were primarily driven by current sensory input. In the detection task, the presence of a target significantly increased the probability of reporting “present” (β = 2.275 ± 0.03, *p* < 0.001). A similarly strong effect of stimulus direction was observed in the motion discrimination task (β = 1.636 ± 0.03, *p* < 0.001), confirming the dominant role of bottom‐up evidence in guiding perceptual reports. In addition to sensory input, participants integrated probabilistic cues into their decisions. In both tasks, higher cue probability increased the likelihood of responding in the expected direction (Experiment 1: β = 0.56 ± 0.02, *p* < 0.001; Experiment 2: β = 0.63 ± 0.02, *p* < 0.001), consistent with flexible incorporation of prior expectations. Responses were also influenced by trial history, although this effect varied across tasks. In the detection task, participants showed a tendency to repeat the previous response (β = 0.89 ± 0.03, *p* < 0.001), indicating a robust choice bias. Conversely, a significant repulsive effect of the previous stimulus was observed (β = –0.19 ± 0.03, *p* < 0.001). This means that participants’ responses are biased away from the previous trial's stimulus. For example, if the target was present in trial_t−1_, they were more likely to report its absence in trial_t_ (and vice versa). In contrast, while a similar repulsive effect of a prior stimulus emerged in the motion task (β = –0.19 ± 0.03, *p* < 0.001), the main effect of previous response was not significant (β = −0.009 ± 0.03, *p* = 0.787), suggesting weaker sequential choice dependencies in this context.

### 3.3 Dimensional Psychosis‐Like Traits Modulate Reliance on Sensory Input and Prior Knowledge

Critically, individual differences in psychosis proneness modulated decision‐making processes in both tasks. Participants with higher PLE scores exhibited reduced reliance on sensory evidence, as reflected in a significant negative interaction between stimulus and PLE (Experiment 1: β = –0.24 ± 0.03, *p* < 0.001; Experiment 2: β = –0.14 ± 0.03, *p* < 0.001). This attenuation suggests that individuals higher in psychosis proneness were less influenced by bottom‐up information, even when the sensory evidence was task‐relevant. Concurrently, the influence of top‐down expectations was amplified in those with higher PLE scores. A significant positive interaction was found between cue probability and PLE in both experiments (Experiment 1: β = 0.07 ± 0.02, *p* < 0.001; Experiment 2: β = 0.17 ± 0.02, *p* < 0.001), indicating that prior expectations had a stronger impact on decision outcomes in individuals with elevated PLE traits. This reciprocal modulation of top‐down and bottom‐up processing provides empirical support for an imbalance in predictive mechanisms underlying perceptual anomalies in psychosis proneness.

### 3.4 Perceptual Threshold Does Not Covary With Psychosis Proneness

Because individual trial‐wise stimulus weighting could in principle be confounded by systematic differences in the stimulus level resulting from adaptive thresholding, we examined whether threshold values covaried with psychosis proneness. In both experiments, there was no evidence that PLE predicted the calibrated threshold (Figure S2, Detection: Threshold ∼ PLE, β = 0.251, *p* = 0.832; Motion discrimination: Threshold ∼ PLE, β = −0.187, *p* = 0.910). Thus, the reduced stimulus impact on choices at higher PLE scores cannot be attributed to high‐PLE participants being tested at systematically different stimulus levels following titration.

### 3.5 SDT Analyses Reveal a General Sensitivity Reduction and Cue‐Dependent Criterion Shifts With Increasing Psychosis Proneness

**FIGURE 3:**
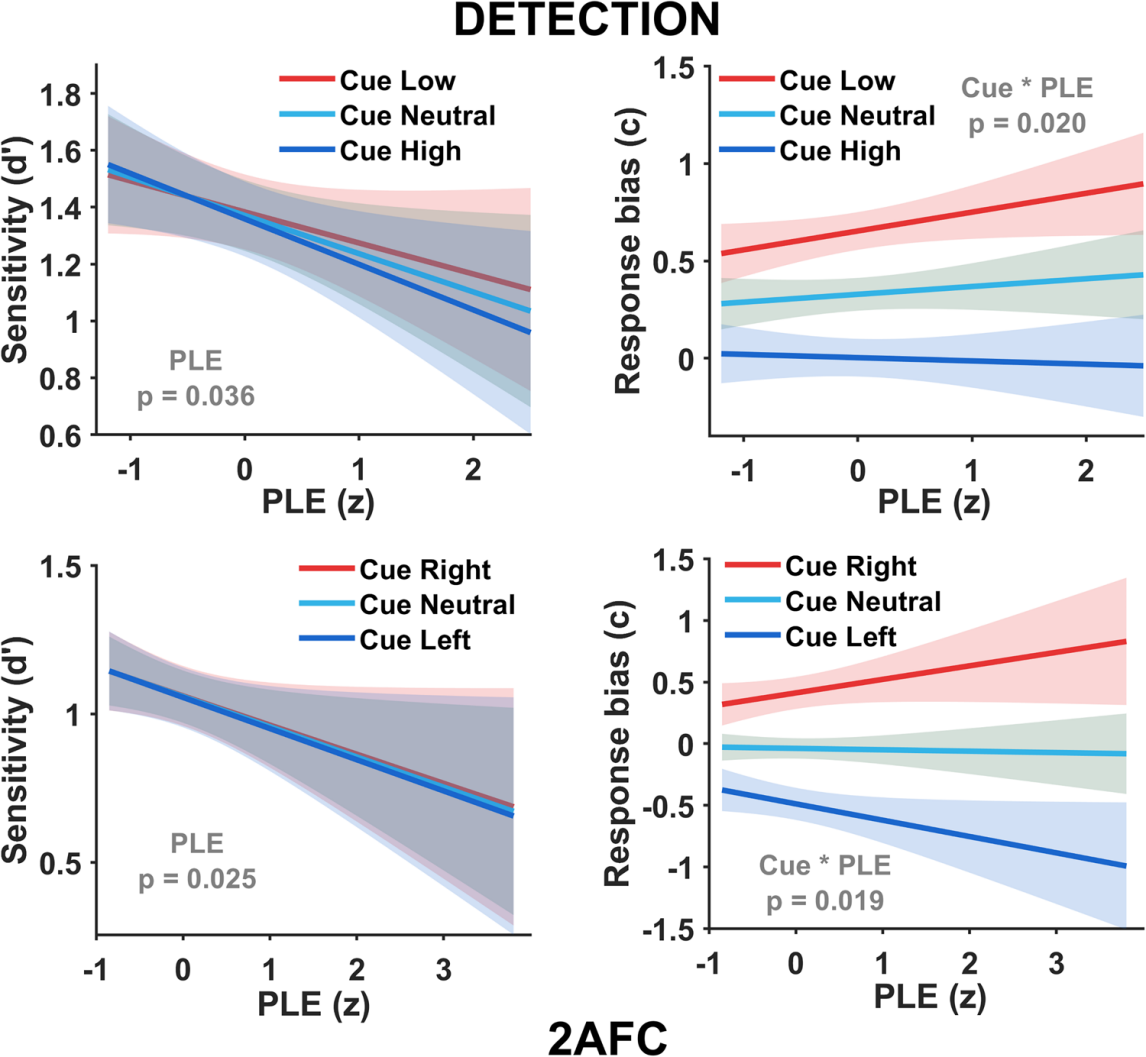
Signal detection indices as a function of psychosis proneness and cue condition in the detection and 2AFC tasks. Top row: detection task; bottom row: motion discrimination (2AFC) task. Left panels depict perceptual sensitivity (d′). Higher values imply a better capacity to distinguish stimulus from noise (in the detection task) and the motion direction of the dots (in the 2AFC). Right panels depict response criterion (c), each plotted as a function of psychosis proneness (PLE, z‐scored) separately for cue conditions (Detection: Cue Low, Neutral, High; Discrimination: Cue Left, Neutral, Right). Negative values imply a liberal criterion (i.e., tendency to report the presence of the target) in the detection task, while in the 2AFC, they imply a tendency toward leftward reporting. Shaded areas represent 95% confidence intervals from mixed‐effects models. Across both tasks, d′ was significantly and negatively associated with psychosis proneness (Detection: β = −0.135, *p* = 0.036; Discrimination: β = −0.102, *p* = 0.025), indicating a general reduction in perceptual sensitivity with increasing PLE. This effect was not modulated by cue condition (Cue × PLE: Detection, *p* = 0.242; Discrimination, *p* = 0.893), consistent with a cue‐independent decrease in discriminability. By contrast, response criterion (c) showed no significant main effect of psychosis proneness (Detection: *p* = 0.353; Discrimination: *p* = 0.787), but a significant Cue × PLE interaction in both tasks (Detection: β = −0.057, *p* = 0.020; Discrimination: β = −0.121, *p* = 0.019). This interaction indicates that individuals with higher psychosis proneness exhibited stronger cue‐dependent shifts in decision criterion toward prior‐consistent responses. Together, these results dissociate a global reduction in sensitivity from an increased prior‐driven modulation of response bias with increasing psychosis proneness. Abbreviations: 2AFC, two‐alternative forced choice; PLE, psychosis‐like experiences.

To further clarify whether psychosis proneness related to perceptual sensitivity versus decision bias, we computed SDT indices (d′ and criterion c) at the subject level separately for each cue condition and tested their association with PLE using mixed‐effects models (Figure 3). Across both experiments, d′ was significantly and negatively associated with psychosis proneness (Detection: β = −0.135, *p* = 0.036; Motion discrimination: β = −0.102, *p* = 0.025), indicating that higher psychosis proneness was associated with reduced perceptual sensitivity. Importantly, this sensitivity reduction was not modulated by cue condition (Detection: Cue * PLE, *p* = 0.242; Motion discrimination: Cue * PLE, *p* = 0.893), suggesting a general, cue‐independent decrease in discriminability with increasing PLE. By contrast, criterion showed no significant main effect of psychosis proneness in either task (Detection: *p* = 0.353; Motion discrimination: *p* = 0.787), indicating that high‐PLE individuals did not adopt a globally different response criterion. However, the Cue × Psychosis interaction on criterion was significant in both experiments (Detection: β = −0.057, *p* = 0.020; Motion discrimination: β = −0.121, *p* = 0.019), revealing that high‐PLE individuals more strongly shifted their decision criterion in the direction of prior expectations. Taken together, these analyses indicate that the negative Stimulus × PLE interaction observed in the trial‐wise GLMM is consistent with a dual mechanism: (a) a cue‐independent reduction in perceptual sensitivity and (b) an amplified cue‐dependent criterion shift. That is, individuals with higher psychosis proneness both detected/discriminated stimuli less accurately and more readily adjusted their decision bias in line with explicit prior expectations.

### 3.6 The Influence of Trial History Depends on Task Context

Interestingly, PLE exerted differential effects on history biases across tasks. In the detection task, PLE did not significantly modulate choice repetition (Choice_t−1_ × PLE: β = 0.026 ± 0.029, *p* = 0.386), but did reduce the repulsive influence of the previous stimulus (Stimulus_t−1_ × PLE: β = 0.075 ± 0.028, *p* = 0.007), suggesting that psychosis‐prone individuals are less likely to bias away from the last trial's sensory input. By contrast, in the motion‐discrimination task, PLE scores were specifically associated with a repulsive bias away from the previous choice (Choice_t−1_ × PLE: β = –0.071 ± 0.033, *p* = 0.033), indicating a reduced tendency to repeat past choices and, instead, a shift toward alternating decisions. No modulatory effect of the previous stimulus was observed (Stimulus_t−1_ × PLE: β = 0.003 ± 0.033, *p* = 0.925), suggesting that, in individuals with high PLE, sequential biases selectively emerge at the decisional level rather than the sensory‐adaptation level in this task. Together, these results reveal task‐dependent signatures whereby psychosis‐like traits alter stimulus‐based adaptation in tasks relying on early sensory encoding and reshape response‐based biases in tasks that depend on higher‐order mechanisms. This pattern highlights task‐contingent differences in the temporal integration of perceptual history, pointing to a hierarchical reorganization in how past information is leveraged to support stable decision‐making over time. To formally test whether PLE‐related modulations of history biases differed across tasks, we conducted a combined GLMM pooling data from both experiments and including Task as a between‐subject factor. Both three‐way interactions were in the predicted direction: PLE‐related attenuation of previous‐stimulus repulsion was more pronounced in the detection task (Task × PLE × Stimulus_t−1_: β = −0.073, SE = 0.044, one‐tail *p* = 0.047), and PLE‐related shift away from choice repetition/increased choice alternation was more pronounced in the discrimination task (Task × PLE × Choice_t−1_: β = −0.096, SE = 0.045, one‐tail *p* = 0.015). Although the interaction with the previous Stimulus reached significance only with a directional test, the consistency of both effects with the a priori predictions and their alignment with the task‐specific simple‐slope pattern (Figure 2) supports a task‐dependent pattern of PLE‐related history modulation.

### 3.7 Control Analysis

To test whether our main effects could be explained by differences in processing speed associated with PLE, we re‐estimated the GLMMs including trial‐wise response time (RT) as an additional covariate. RT did not significantly predict choice in either task (Detection: β = 0.029, *p* = 0.161; Discrimination: β = 0.073, *p* = 0.249). Crucially, the key PLE interaction terms were essentially unchanged and remained significant after controlling for RT (all *p*s ≤ 0.034; see Supplementary Material). This indicates that the PLE‐related modulation of sensory evidence, cue use, and history effects is not accounted for by differences in processing speed. Moreover, because raw PLE scores differed between the two samples (with higher values in the detection task and a wider upper range), we ran an additional sensitivity analysis to ensure that the effects found in the detection task were not driven by the small subset of detection participants whose PLE scores exceeded the maximum observed in the discrimination sample. We refit the detection‐task GLMM after excluding participants with PLE > 16 (80 → 76 subjects). The key PLE effects remained significant and went in the same direction as in the full dataset (Stimulus_t_ × PLE β = −0.153, *p* < 0.001, Cue_t_ × PLE β = 0.063, *p* < 0.001; Stimulus_t−1_ × PLE: β = 0.077, *p* = 0.011), indicating that the reported PLE‐related effects in the detection task are not driven by participants with extreme PLE scores.

### 3.8 Task‐Specific Computational Signatures of PLE Subdimensions

To examine whether distinct facets of psychosis proneness show dissociable computational signatures, we repeated the main GLMM analyses using each positive SPQ subscale separately in place of the composite PLE score (see Supplementary Material and Table S1 for full model outputs). Across tasks, MT most consistently reproduced the composite pattern: higher MT was associated with reduced stimulus weighting (negative Stimulus_t_ * MT interaction) and increased cue‐based bias (positive Cue_t_ * MT interaction) in both detection and discrimination. MT also showed a broadly similar task‐dependent history profile, with significant modulation of stimulus history in the detection task and a trend‐level modulation of choice history in the discrimination task. In contrast, UPE showed a comparatively detection‐weighted profile. UPE was associated with a stronger reduction in the influence of current sensory evidence in detection than in discrimination, and with more pronounced modulation of sensory‐history terms in detection. Notably, UPE did not show evidence for a general increase in cue reliance in the discrimination task; cue‐related effects were instead evident in the detection context. Finally, IoR showed a comparatively discrimination‐weighted profile, characterized by stronger modulation of choice history terms and cue‐related influences in the discrimination task relative to detection. This pattern is consistent with the possibility that IoR preferentially relates to higher‐order decision dynamics, whereas UPE is more tightly linked to sensory‐dominant contexts.

## 4 Discussion

Our findings show that PLEs shape perceptual decision‐making by altering the balance between sensory evidence, explicit probabilistic cues, and history‐dependent sequential biases. Across two complementary tasks—detection under uncertainty and motion‐direction discrimination—elevated psychosis proneness was associated with reduced stimulus weighting, stronger reliance on instructed cue information, and task‐dependent alterations in how recent stimulus and choice history influence decisions. These results converge with predictive processing accounts proposing that disruptions in hierarchical precision‐weighting underlie key phenomena along the psychosis continuum [10, 16, 41].

Across both tasks, choice behavior was positively influenced by current sensory input (Stimulus_t_) and cue probability (Cue_t_), indicating that perceptual decisions reflect the joint contribution of bottom‐up evidence and top‐down explicit expectations. A consistent repulsive effect of the previous stimulus (Stimulus_t__−__1_) also emerged, suggesting a history‐dependent mechanism that biases decisions away from recent input. Notably, only in the detection task, the previous choice (Choice_t−1_) significantly influenced current decisions, revealing a tendency toward choice repetition that was absent in the motion discrimination task. These patterns suggest shared mechanisms for integrating evidence and prior information, alongside task‐specific differences in response history effects. Crucially, the strength and direction of these influences were differentially modulated by PLE, affecting the balance between sensory evidence, prior information, and choice history.

First, higher PLE scores were associated with a reduced influence of current sensory evidence on choice. This effect was robust across both the detection and motion discrimination tasks. These findings resonate with prior work in schizophrenia and schizotypy indicating diminished weighting of bottom‐up evidence during perceptual inference [14, 16, 34, 42]. Importantly, our results support the view that alterations in sensory evidence weighting are continuous across the psychosis spectrum [12]. In line with canonical accounts of perceptual decision‐making, lower PLE scores predicted greater reliance on current sensory evidence than higher PLE scores, consistent with the idea that sensory input should be weighted more strongly when it provides informative evidence for the decision. Crucially, complementary SDT analyses corroborate and refine this finding. Across both tasks, higher PLE scores were associated with significantly lower d′, and this sensitivity reduction was not modulated by cue condition, indicating a general, cue‐independent decrease in perceptual discriminability. This indicates that the reduced stimulus weighting observed in the trial‐wise GLMM is partly consistent with a reduction in the ability to extract signal from noise, not merely a shift in response strategy. At a mechanistic level, this effect could reflect atypical processing in early sensory processing regions or altered functional connectivity with higher‐order associative areas implicated in predictive representation [43, 44, 45]. Consistent with this possibility, previous neuroimaging research has documented reduced sensory cortical activation alongside increased top‐down modulatory influence from frontal cortical regions in schizophrenia and schizotypy [4, 43, 46]. Moreover, both schizophrenia and subclinical schizotypy have been associated with a lower individual alpha frequency [47, 48, 49, 50, 51], a neural marker closely linked to the enhanced resolution of sensory evidence sampling [52, 53, 54, 55, 56, 57, 58]. This suggests that the diminished reliance on sensory input observed in individuals with elevated PLEs may partly stem from disruptions in alpha oscillatory dynamics.

A complementary yet distinct finding was the enhanced reliance on explicit, instructed probabilistic cues in participants with elevated psychosis‐like traits. Regardless of the PLE scores, the weight assigned to the cue was robust across tasks. However, the magnitude of the cue‐dependent biases diverged: low‐PLE individuals exhibited moderate prior‐dependent effects, whereas high‐PLE individuals showed markedly stronger reliance on priors. This behavioral pattern is further supported by SDT criterion analyses: the Cue × PLE interaction on criterion was significant in both tasks, indicating that high‐PLE individuals more strongly shifted their decision criterion in the direction of prior expectations. Thus, the enhanced cue reliance observed in the GLMM is mirrored by a greater cue‐dependent criterion adjustment in the SDT framework. This pattern further supports predictive‐processing accounts in which psychosis‐spectrum traits involve altered precision‐weighting, such that expectations exert a stronger influence on choice relative to sensory evidence under uncertainty [3, 41]. Notably, because higher PLE was also associated with lower d′, increased cue reliance may partly reflect adaptive reweighting when sensory evidence is less precise, rather than stronger priors in an absolute sense.

Then, we examined whether PLEs modulate history‐dependent bias. In a seminal set of studies, Fritsche et al. [59] showed, using separate orientation‐estimation tasks, that perceptual reports are typically repelled from the previous stimulus, whereas choices exerted an attractive force. This dissociation was subsequently refined by Bosch et al. [60], who demonstrated that sensory history and choice history can bias perceptual experience in opposite directions while jointly shaping behavior. Building on this framework, our study suggests that these dissociable history components are differentially altered as a function of psychosis proneness, and that the direction and strength of these alterations depend on task demands and on the specific computational processes recruited by the context. In the detection task, higher PLE scores were not associated with changes in response repetition but were linked to an attenuation of the repulsive bias from the previous stimulus—indicating a reduced influence of recent sensory (but not choice) history on behavior. In contrast, in the motion discrimination task, high‐PLE scores were associated with a significant repulsive bias relative to their previous choices, suggesting a shift from choice persistence toward reduced persistence/increased alternation, while the effect of the previous stimulus on current decisions remained unaffected by PLE scores. These different signatures underscore that the influence of psychosis proneness on choice history is not uniform, but varies as a function of the inferential demands imposed by different task structures.

This divergence cannot be explained by differences in task difficulty, as both tasks were calibrated to yield comparable accuracy (∼70%). Rather, it likely reflects differences in sensory and decision architecture between the two decisional contexts. Indeed, it is possible that the two tasks hinge on different levels of the perceptual hierarchy. In the detection task, repulsive effects from previous stimuli could be interpreted as reflecting low‐level sensory adaptation—a form of neural gain control that suppresses responses to recently encountered features. Adaptation effects might be a consequence of one important goal of the visual system, that is, to maximize sensitivity to changes in the physical environment [59]. Our finding that this repulsion is selectively attenuated in high‐PLE individuals suggests a deficit in early sensory mechanisms. This aligns with evidence of impaired adaptation and reduced gain control in schizophrenia‐spectrum conditions [61, 62] and suggests that subclinical traits may already manifest as reduced suppression of prior sensory traces. This interpretation could also fit with the highlighted robust negative interaction between stimulus in the current trial and PLE traits: individuals with elevated psychosis proneness tend to down‐weight actual sensory input when forming a decision. Consequently, the sensory representation itself may be too underweighted to elicit a reliable adaptive response, further dampening the typical repulsive after‐effects associated with recent stimulation.

**FIGURE 4:**
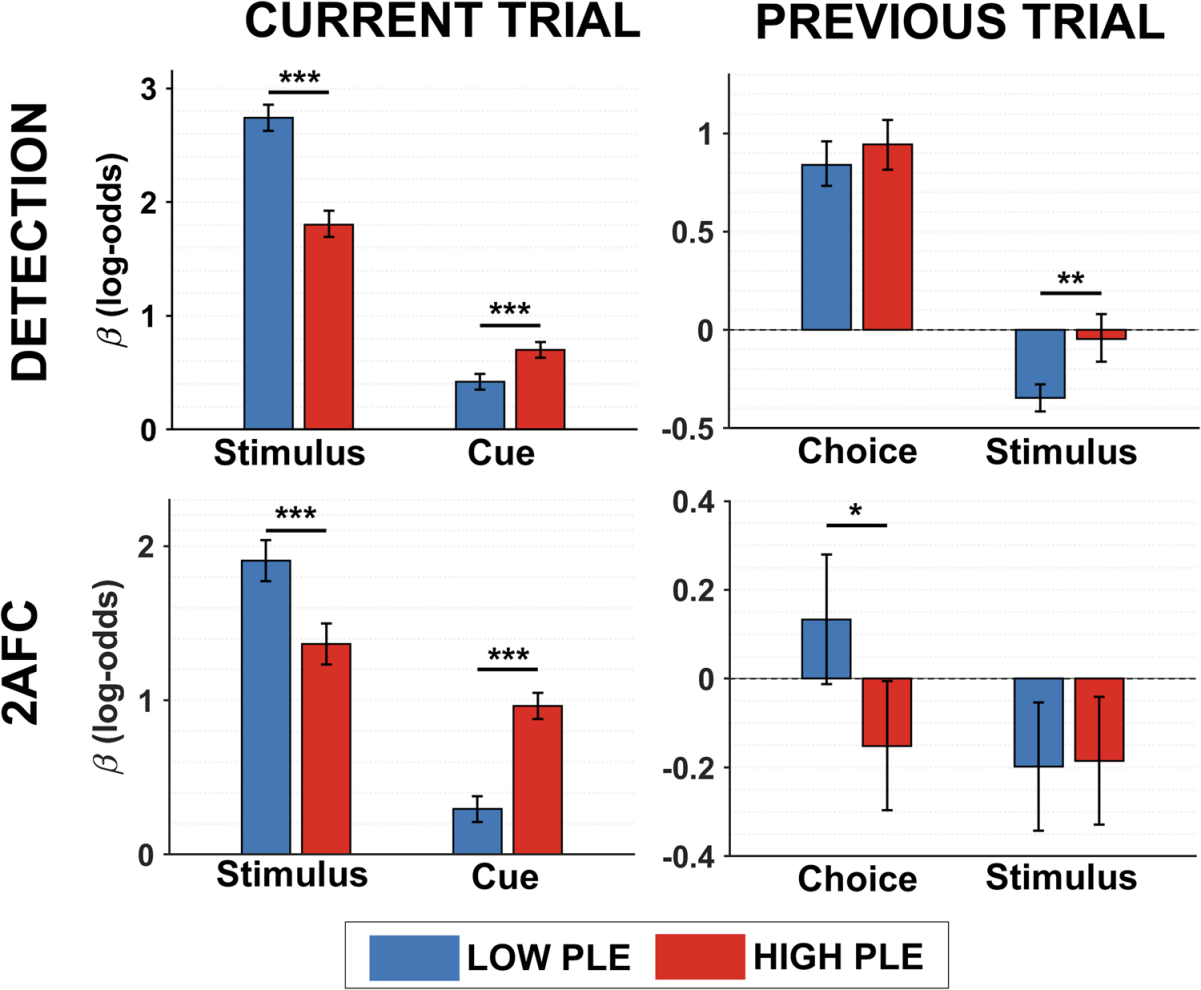
Task‐dependent modulation of perceptual decision‐making by psychosis‐like experiences. Conditional β coefficients (log‐odds scale) from generalized linear mixed‐effects models for the detection task (top row) and Motion Discrimination task (bottom row), evaluated at low and high psychosis proneness (−2 and +2 SD of the PLE regressor). Bars reflect model‐estimated slopes (log‐odds). Left panels show effects of current trial predictors: Current Stimulus reflects the influence of current sensory evidence, and CUE reflects the influence of explicit cue‐induced expectations. Right panels show history effects: Previous Choice (Choice_t−1_) captures choice repetition or alternation biases, while Previous Stimulus (Stimulus_t−1_) captures stimulus‐driven repulsive or attractive serial dependencies. Error bars represent ±1.96 standard errors derived via the delta method. Horizontal brackets with asterisks denote statistically significant Predictor × Psychosis interactions (* *p* < 0.05, ** *p* < 0.01, *** *p* < 0.001), indicating differential modulation by PLE. In these GLMM estimates, higher PLE scores are associated with (1) reduced weighting of current sensory evidence (Stimulus), (2) enhanced reliance on cue‐based expectations (Cue), (3) attenuated repulsive stimulus‐history effects in detection (Previous Stimulus), and (4) a shift away from choice repetition/increased choice alternation in motion discrimination (Previous Choice). Abbreviations: 2AFC, two‐alternative forced choice; PLE, psychosis‐like experiences.

By contrast, in the motion discrimination task—where perceptual decisions depend on the accumulation of noisy evidence over time—a different form of bias emerges. High‐PLE participants exhibit a significant repulsive tendency from their own previous response, being prone to shift away from their last choice. In contrast, model‐implied slopes suggest comparatively greater choice persistence at low PLE (Figure 4), even though the average main effect of previous choice was not significant at the group level, highlighting that PLE primarily modulates the direction of the choice‐history tendency rather than simply scaling a uniform repetition bias. One possible interpretation is that psychosis‐prone individuals adopt a compensatory strategy at the decisional level, actively avoiding response repetition. While this pattern could reflect a strategic avoidance of repetition under uncertainty, it may also align with a broader literature linking psychosis‐spectrum traits to increased choice‐switching and reduced choice persistence (e.g., “win‐switching” or heightened behavioral volatility in reinforcement‐learning settings [63, 64]). In this framework, the negative Choice _t−1_ × PLE effect may reflect reduced stability of internal choice policies and/or an increased subjective estimate of environmental volatility. Consistent with this account, increased belief instability has been reported in psychotic disorders [65], and increased inferences about context change have been documented in schizophrenia [66]. Such an overestimation may lead psychosis‐prone individuals to infer that the correct response on the next trial is likely to differ from the previous one. Crucially, this different effect—reduced stimulus‐driven repulsion in the detection task versus decision‐level repulsion in the motion task—mirrors prior evidence for two distinct history‐bias mechanisms: a fast, repulsive sensory adaptation mechanism and a slower, attractive bias toward previous trials, reflecting decision‐level stability [59]. Our findings suggest that psychosis proneness not only dampens the former but appears to invert the latter—shifting toward repulsion in the discrimination task.

Moreover, Fritsche et al. [59] demonstrated that the attractive decision bias likely originates from a post‐perceptual shift in working memory representations. This is consistent with earlier reports of trial‐to‐trial carryover effects in short‐term memory [67]. The inversion observed in high‐PLE individuals may also reflect a deficit in maintaining stable representations of prior decisions, possibly due to reduced working memory (WM) capacity [68], which in turn would lead to decreased reliance on information from the previous trial. Crucially, Xie et al. [69] showed that individuals with more schizotypal features retained less precise representations in visual WM without a significant reduction in the number of retained WM representations.

Motivated by the possibility that different facets of psychosis proneness may map onto partially distinct computational signatures, we additionally examined the three SPQ subscales contributing to our composite PLE measure (MT, UPE, IoR). MT largely mirrored the composite results across both tasks, showing the same directional pattern for the key interactions (reduced stimulus weighting and increased cue reliance) along with a broadly similar profile for history‐dependent terms. By contrast, UPE showed a more detection‐weighted pattern, with comparatively stronger associations with stimulus‐ and stimulus‐history–related terms, whereas IoR showed a more discrimination‐weighted pattern, most clearly expressed in cue‐related and choice‐history–related terms. This effect is consistent with the idea that psychosis‐spectrum traits may differentially impact sensory‐dominant versus decision‐dominant components of inference and may differentially relate to explicit, instructed expectations versus history‐derived influences.

While this study offers important insights, several considerations merit acknowledgment. First, although we examined psychosis proneness dimensionally, future research could benefit from longitudinal designs to understand whether observed computational alterations predict transition from subclinical PLEs to clinical psychosis symptoms. Additionally, integrating neuroimaging methods could further clarify the neural substrates underlying enhanced explicit cue reliance observed here, particularly regarding connectivity between early sensory and high‐level predictive processing regions. Moreover, we did not collect formal measures of general cognitive ability (e.g., IQ), although there is a modest association between PLEs and circumscribed cognitive impairment [70]; future work should test whether the reported PLE‐related modulations remain after controlling for cognitive capacity. A further limitation concerns the stimulus‐response mapping, which was fixed within each task. Although cue meaning and validity were explicitly instructed, fixed mappings may allow cue‐driven response preparation to contribute to behavior. Our interpretation, therefore, focuses on cue‐induced expectation effects at the level of choice, while acknowledging that decisional and motor components cannot be fully dissociated in the current design. Future work should orthogonalize mapping (e.g., counterbalanced keys across participants or blocks) to separate expectation effects from associative or motor contributions more directly. Furthermore, examining the generalizability of our findings across sensory modalities, task structure, and cognitive domains remains a critical direction for future research. While our results consistently indicate reduced sensory weighting and enhanced cue reliance with increasing PLE, aligning with several lines of evidence [3, 4, 8, 14, 15, 17, 46, 71, 72, 73, 74, 75], a body of research points in the opposite direction. Goodwin et al. [76], using a perceptual decision‐making task with Bayesian modeling across two large samples, reported that PLEs were associated with overweighting of sensory information relative to prior expectations, driven by decreased precision afforded to priors. Stuke et al. [77] found that delusion proneness was associated with reduced reliance on priors in a random‐dot motion paradigm. In the auditory domain, Schaub et al. [78] reported increased reliance on sensory evidence and reduced weighting of explicit, probabilistic prior with higher psychosis proneness, again the opposite pattern to ours. Notably, Eckert et al. [22] found that, while participants with higher psychosis proneness showed increased reliance on probabilistic priors in a visual discrimination task akin to the one employed in our design, no such effect emerged when the same probabilistic structure was applied to auditory stimuli, highlighting additional modality‐specific variability. Several factors may contribute to these discrepant findings. First, differences in how sensory evidence is manipulated likely play a central role. Studies reporting increased sensory weighting have generally varied stimulus evidence across trials (e.g., varied coherence levels), enabling direct estimation of how strongly participants rely on sensory input relative to priors. By contrast, our design held sensory evidence constant at each participant's perceptual threshold, which effectively isolates prior influences on choice but precludes formal testing of stimulus‐strength × PLE interactions. It remains possible that graded stimulus manipulations would reveal more nuanced patterns. Second, modality‐specific differences in the balance between feedforward and feedback processing may lead to divergent PLE‐related effects on evidence weighting in visual versus auditory perception. Third, differences in the specific facets of psychosis proneness captured by each study's measure, ranging from the SPQ subscales used here to the delusions inventory [79] and the Cardiff anomalous perception scale [80] may further contribute, as different instruments may tap into partially distinct computational phenotypes. Future studies systematically varying evidence levels within the same paradigm would complement our findings by characterizing whether high‐PLE individuals show altered precision weighting of graded sensory signals. Finally, the two experiments were conducted in independent samples that differed significantly in their raw PLE scores, with higher mean PLE scores in the detection task sample than in the motion‐discrimination sample. Although PLE scores were z‐scored within each sample before fitting the combined GLMM, and although the detection‐task effects remained robust after excluding participants with PLE scores above the maximum observed in the discrimination sample, this between‐sample difference constrains the interpretation of cross‐task comparisons. The task‐dependent pattern of PLE‐related history effects should, therefore, be interpreted cautiously and confirmed in future studies testing both tasks within the same participants.

Overall, our work fits with the influential view that psychosis‐related effects do not map onto a single “strong‐prior” or “weak‐prior” axis. Predictive‐processing accounts increasingly frame psychosis as precision dysregulation that is both hierarchical‐level–specific and source‐specific [6, 7, 41]. At lower levels, some paradigms reveal weakened perceptual priors, expressed as diminished percept stabilization or increased sensitivity to disambiguating evidence [4, 81, 82]. At higher levels, other findings highlight overly stable or overly precise beliefs, including the enhanced reliance on explicit cues observed here and in previous work [3, 10, 14, 15]. This apparent tension can be accommodated within hierarchical frameworks in which weak low‐level priors coexist with stronger high‐level beliefs. Within this landscape, our results point to a selective amplification of high‐level, cue‐induced prior expectations under matched sensory ambiguity, coupled with a general alteration of low‐level, history‐dependent influences. Moreover, by revealing different disruptions in how sensory evidence, prior information, and choice history are integrated across contexts, our results point to potential targets for cognitive or neuromodulatory interventions [58, 83]. Approaches aimed at restoring the balance between external evidence and internal expectations—such as modulating the weighting of prior beliefs—may hold promise in mitigating early perceptual disturbances along the psychosis continuum.

## 5 Conclusion

Our study shows that psychosis‐like traits modulate perceptual decision‐making, characterized by reduced weighting of current sensory evidence, increased reliance on explicit probabilistic cues, and task‐dependent signatures in serial dependence. Together, these results support a nuanced view in which different sources of prior information (explicit vs. history‐derived) are differentially expressed along the psychosis continuum. Rather than implying a unitary “strong‐” or “weak‐prior” phenotype, our findings are compatible with hierarchical and source‐specific accounts of altered inference, where the relative contribution of bottom‐up evidence, instructed expectations, and recent history varies with task demands. By showing that these signatures are detectable in subclinical variability and depend on the inferential structure of the task, our findings underscore the need for multidimensional frameworks to characterize psychosis vulnerability across perceptual contexts, and motivate future work testing their generalization to clinical populations and neural mechanisms.

## Author Contributions

Conceptualization, L.T. and V.R. Methodology, L.T. and V.R. Formal analysis, L.T. Investigation, L.T. Supervision, V.R. Writing – original draft preparation, L.T. and V.R. Writing – review and editing, V.R. and L.T.

## Conflicts of Interest

The authors declare that they have no financial conflicts of interest or personal connections that could have potentially impacted the work presented in this paper.

## Supporting information




**Supplementary Material**: nyas70347‐sup‐0001‐SuppMat.docx

## Data Availability

All data and code used in this study are openly available on the Open Science Framework (OSF): https://osf.io/x4tjp/.
