# Supplementary material for: Predictive Inference Alterations in Psychosis Proneness Show Task‐Dependent Signatures
Source: Ann N Y Acad Sci. 2026 Jul 24;1561(1):e70347. doi: 10.1111/nyas.70347 (PMC13399831; doi:10.1111/nyas.70347)
Supplement: Supplementary file 1 — Supplementary Material: nyas70347‐sup‐0001‐SuppMat.docx [file NYAS-1561-0-s001.docx]

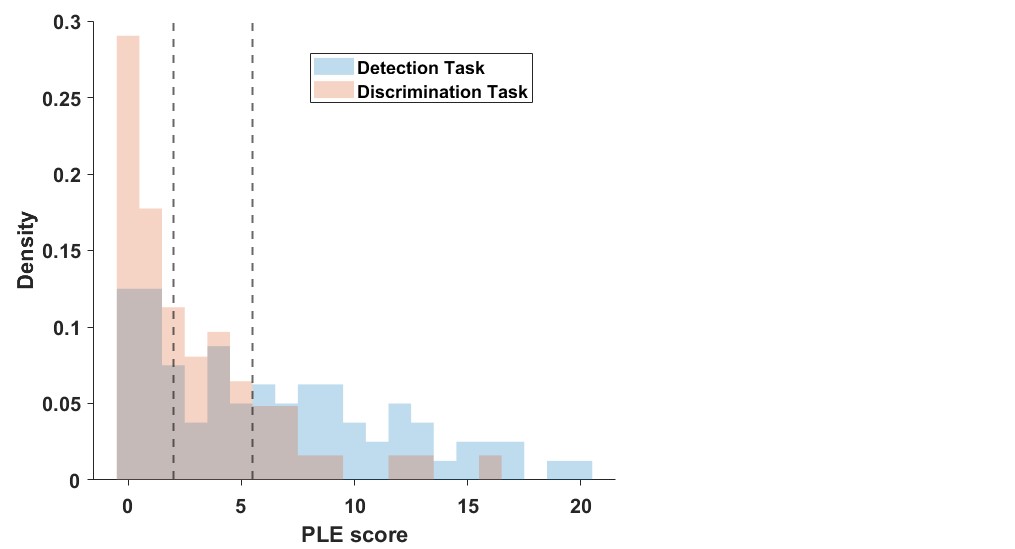


**Figure S1. Distribution of psychosis-like experiences (PLE) scores across tasks**

Psychosis-like experiences (PLE) composite scores (sum of the SPQ subscales *Magical Thinking*, *Ideas of Reference*, and *Unusual Perceptual Experiences*) are shown separately for the Detection task (N = 80) and the Random Dot Motion (RDM) discrimination task (N = 62). The figure illustrates the full score distributions; summary statistics are reported below for reference: Detection, mean ± SD = 6.38 ± 5.33, median [Q1–Q3] = 5.50 [1.50–10.00], range = 0–20; RDM, mean ± SD = 2.92 ± 3.44, median [Q1–Q3] = 2.00 [0.00–4.00], range = 0–16.


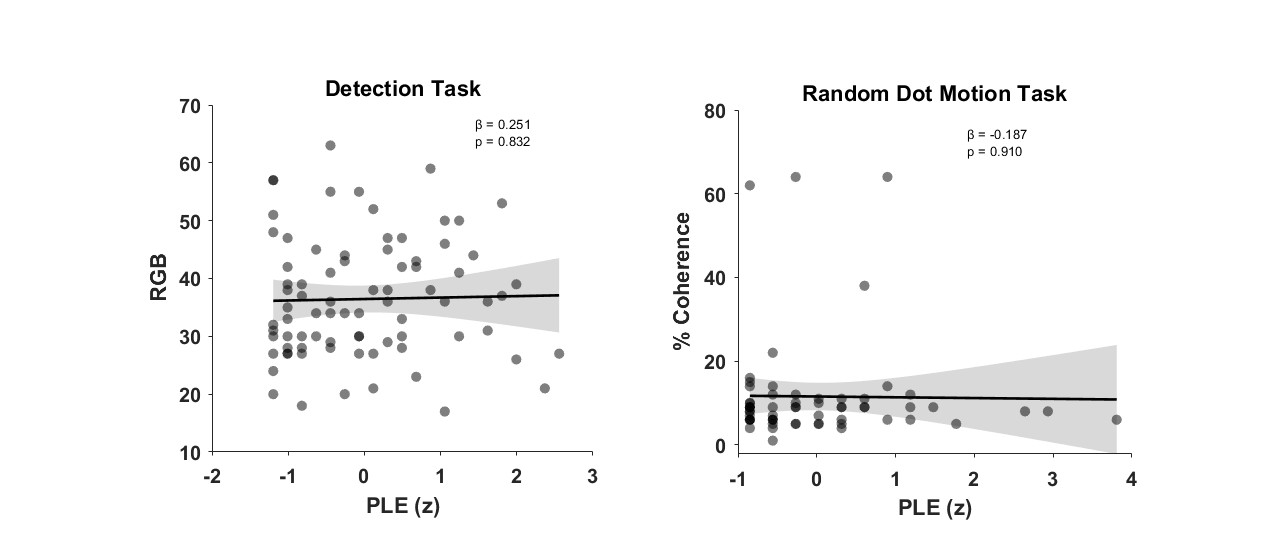


**Figure S2. Relationship between psychosis-like experiences and perceptual thresholds across tasks.** (A) Detection task: Scatterplot showing individual perceptual thresholds (RGB values) as a function of standardized psychosis-like experience scores. Detection thresholds are expressed as the RGB value of the grey circle on the white background (with the corresponding value on the black background covarying accordingly), per the titration procedure. Each dot represents one participant. The black line represents the linear regression fit, with shaded area indicating 95% confidence interval. Higher psychosis scores were associated with no change in detection thresholds (β = 0.25, p = 0.83). (B) Random dot motion task: Perceptual thresholds expressed as percentage of coherent motion required for discrimination. Layout and conventions as in panel A. The relationship between psychosis-like experiences and motion coherence thresholds was non-significant (β = -0.19, p = 0.91).

**Subscale follow-up analyses**

To assess whether the effects observed with the composite PLE score were attributable to a specific facet of psychosis-proneness, we conducted exploratory follow-up GLMMs in which the composite score was replaced by each constituent SPQ subscale: Magical Thinking, Ideas of Reference, and Unusual Perceptual Experiences (each z-scored within sample). Model structure, predictors, and random-effects specification were otherwise identical to the primary analyses. Interpretation of history terms is anchored to the sign of the corresponding main history effect within each task (repulsive vs attractive), as reported in the primary GLMM. For ease of comparison with the main text, we reported only the trait-by-predictor interaction terms from each subscale model, separately for the detection and motion discrimination tasks.

- Sensory evidence (Stimulus × Trait)

Across both tasks, the interaction between current sensory evidence and each subscale remained negative and significant, indicating that the reduced influence of the current stimulus on choice was not specific to a single subcomponent of the composite score (Detection: Ideas of Reference β = −0.134, p < .001; Magical Thinking β = −0.194, p < .001; Unusual Perceptual Experiences β = −0.263, p < .001; Discrimination: Ideas of Reference β = −0.134, p < .001; Magical Thinking β = −0.130, p < .001; Unusual Perceptual Experiences β = −0.062, p = .038).

- Explicit expectations (Cue × Trait)

Cue-related modulation showed a more differentiated profile across subscales. In the motion discrimination task, Cue × Trait effects were most evident for Ideas of Reference (β = 0.187, p < .001) and Magical Thinking (β = 0.246, p < .001), whereas Unusual Perceptual Experiences showed no reliable cue interaction (β = 0.009, p = .633). In the detection task, cue interactions were present for Magical Thinking (β = 0.112, p < .001) and Unusual Perceptual Experiences (β = 0.066, p < .001), but not for Ideas of Reference (β = 0.004, p = .803). Together, these results suggest that cue reliance in the discrimination task is more consistently associated with referential/delusion-like dimensions, whereas Unusual Perceptual Experiences does not show a robust cue-related signature in that context.

- History-dependent influences (*Stimulus _t-1_ ×* Trait; Choice *_t-1_* × Trait)

History terms again revealed a task-dependent pattern. In Detection task, Higher Unusual Perceptual Experiences scores were associated with a positive Stimulus *_t-1_* × Trait interaction (β = 0.076, p = .0067), and a similar pattern was observed for Magical Thinking (β = 0.066, p = .0219), whereas Ideas of Reference showed a trend-level modulation (β = 0.047, p = .091). Given that the main Stimulus *_t-1_* effect is repulsive in this task, a positive interaction indicates an attenuation of repulsive stimulus-history carry-over. Interactions with Choice _t-1_ were small and not reliable across subscales (all ps ≥ 0.205). In contrast, in Motion discrimination task the modulation of choice history (Choice _t-1_ × Trait) was most pronounced for Ideas of Reference (β = −0.142, p < 0.001), with marginal support for Magical Thinking (β = −0.064, p = 0.061), and no robust association for Unusual Perceptual Experiences (β = 0.035, p = 0.285). This pattern is consistent with the view that referential/delusion-like dimensions more selectively relate to alterations in choice persistence/alternation in a task that relies on evidence accumulation and decision-level dynamics.

- Formal tests of task specificity (Task × Subscale × Predictor)

To directly test whether these subscale effects differed between tasks, we also fit combined models including Task as a between-subject factor and evaluated Task × Subscale × Predictor interactions. These models supported the descriptive task differences. For Ideas of Reference, the task specificity was confirmed for both cue reliance (Cue × Reference × Task: p < .001) and choice-history modulation (Choice _t-1_ × Reference × Task: p < .001), whereas stimulus-related effects did not differ across tasks (Stimulus × Reference × Task: p = 0.99). For Magical Thinking, task differences were also evident for cue effects (Cue × MagicalThinking × Task: p < .001) and choice-history modulation (Choice _t-1_ × MagicalThinking × Task: p = 0.026), while the stimulus interaction showed no reliable task moderation (Stimulus_t_ × MagicalThinking × Task: p = 0.12). For Unusual Perceptual Experiences, task specificity was strongest for stimulus-related terms (Stimulus × UPE × Task: p < .001) and stimulus-history (Stimulusₜ₋₁× UPE × Task: p = 0.0042), consistent with a preferential association with sensory and stimulus-history components in the detection task; additionally, cue modulation differed by task (Cue × UPE × Task: p = 0.0195).

Overall, these exploratory subscale analyses provide a finer-grained view of the composite PLE effects without changing their interpretation: all three subscales contributed to reduced stimulus weighting, while Ideas of Reference and Magical Thinking more consistently tracked cue and/or choice-history effects in discrimination, while Unusual Perceptual Experiences and Magical Thinking more selectively tracked stimulus-history attenuation in detection.

| Table S1. Subscale × Predictor interactions | | | | | | | |
| --- | --- | --- | --- | --- | --- | --- | --- |
| *Each SPQ subscale (z-scored within sample) replaced the composite PLE score in the main GLMM. Only trait × predictor interactions are shown.* | | | | | | | |
|  |  |  |  |  |  |  |  |
| Subscale | **Interaction** | **β (Det)** | **p (Det)** | **Sig** | **β (RDM)** | **p (RDM)** | **Sig** |
| *Ideas of Reference* | Stimulus_t_ × IoR | -0.134 | < .001 | *** | -0.134 | < .001 | *** |
|  | Cue_t_ × IoR | 0.004 | 0.803 |  | 0.187 | < .001 | *** |
|  | Choice _t-1_ × IoR | 0.025 | 0.393 |  | -0.142 | < .001 | *** |
|  | Stimulus _t-1_ × IoR | 0.047 | 0.091 | † | 0.036 | 0.274 |  |
| *Magical Thinking* | Stimulus_t_ × MT | -0.194 | < .001 | *** | -0.130 | < .001 | *** |
|  | Cue_t_ × MT | 0.112 | < .001 | *** | 0.246 | < .001 | *** |
|  | Choice _t-1_ × MT | 0.038 | 0.205 |  | -0.064 | 0.061 | † |
|  | Stimulus _t-1_ × MT | 0.066 | 0.022 | * | 0.021 | 0.536 |  |
| *Unusual Perceptual Experiences* | Stimulus_t_ × UPE | -0.263 | < .001 | *** | -0.062 | 0.038 | * |
|  | Cue_t_ × UPE | 0.066 | < .001 | *** | 0.009 | 0.633 |  |
|  | Choice _t-1_ × UPE | -0.002 | 0.958 |  | 0.035 | 0.285 |  |
|  | Stimulus _t-1_ × UPE | 0.076 | 0.007 | ** | -0.046 | 0.152 |  |
| *Note. Det = Detection task; RDM = Random Dot Motion discrimination task. β = standardized coefficient from GLMM. All p-values are two-tailed. *** p < .001, ** p < .01, * p < .05, † p < .10.* | | | | | | | |

**Supplementary Results: Control analyses including response time (RT)**

To evaluate whether the observed PLE effects on perceptual choices might reflect differences in processing speed, we re-ran the main GLMMs for each task including trial-wise response time (RT) as an additional fixed-effect covariate. RT was not a significant predictor of choice in either paradigm (Detection: β = 0.029, p = 0.161; Discrimination: β = 0.073, p = 0.249). Importantly, all theoretically relevant PLE interaction terms remained statistically reliable and closely matched the original estimates:

Detection task

- Stimulus_t_ * PLE: β = −0.243, p < .001
- Cue * PLE: β = 0.073, p < .001
- Stimulus _t-1_ * PLE: β = 0.073, p = 0.010

Discrimination task

- Stimulus _t_ * PLE: β = −0.135, p < .001
- Cue * PLE: β = 0.165, p < .001
- Choice _t-1_ * PLE: β = −0.071, p = 0.034

**Group-based Tercile analysis**

To complement the dimensional analysis based on continuous PLE scores, we conducted an exploratory analysis in which participants were stratified into terciles according to their composite PLE scores. Individuals in the lowest and highest terciles were assigned to Low and High PLE groups, respectively. In the detection task, the Low-PLE group had a mean composite score of *M* = 1.07 (SD = 1.00), whereas the High-PLE group had a mean score of *M* = 12.81 (SD = 3.24). In the motion discrimination (2AFC) task, the Low-PLE group had a mean composite score of *M* = 0.38 (SD = 0.49), and the High-PLE group had a mean score of *M* = 6.71 (SD = 3.30). A generalized linear mixed-effects model (GLMM) was then fitted separately for each task, using the same fixed-effect structure as the main model but replacing the continuous PLE predictor with a binary group factor (PLEgroup: Low = 0, High = 1). Fixed effects included current stimulus (Stimulusₜ), previous choice (Choice _t-1_), previous stimulus (Stimulus _t-1_), cue probability (Cue _t_), and their interactions with PLEgroup; random intercepts were specified for each participant. The tercile-based analysis largely recapitulated the continuous effects. In the detection task (Experiment 1), High-PLE participants showed a reduced impact of current sensory evidence relative to Low-PLE participants (Stimulus _t_ × PLEgroup: β = −0.462 ± 0.060, *p < .001*), together with a stronger cue-driven bias (Cue _t_ × PLEgroup: β = 0.159 ± 0.037, *p < .001*). PLEgroup did not significantly modulate choice repetition (Choice _t-1_ × PLEgroup: β = −0.098 ± 0.070, p = 0.163). Crucially, stimulus-history repulsion was attenuated in High-PLE participants (Stimulus _t_₋₁ × PLEgroup: β = 0.191 ± 0.068, p = 0.0046), consistent with a reduced bias away from the previous stimulus.

In the motion discrimination task (Experiment 2), High-PLE participants again showed reduced sensory weighting (Stimulus _t_ × PLEgroup: β = −0.193 ± 0.065, p = 0.0031) and increased cue reliance (Cue _t_ × PLEgroup: β = 0.124 ± 0.042, p = 0.0033). In addition, they showed reduced choice persistence / increased alternation relative to Low-PLE participants (Choice _t-1_ × PLEgroup: β = −0.273 ± 0.073, *p < .001*). The modulation of stimulus-history effects by PLEgroup was not significant in this task (Stimulus _t_₋₁ × PLEgroup: β = 0.109 ± 0.072, p = 0.131). Together, these findings indicate that elevated psychosis-like traits are associated with a shift in the balance between bottom-up evidence and explicit probabilistic expectations across tasks, whereas history-dependent modulations remain task-dependent: detection shows an attenuation of stimulus-history repulsion, whereas discrimination shows a shift in choice-history weighting toward alternation.


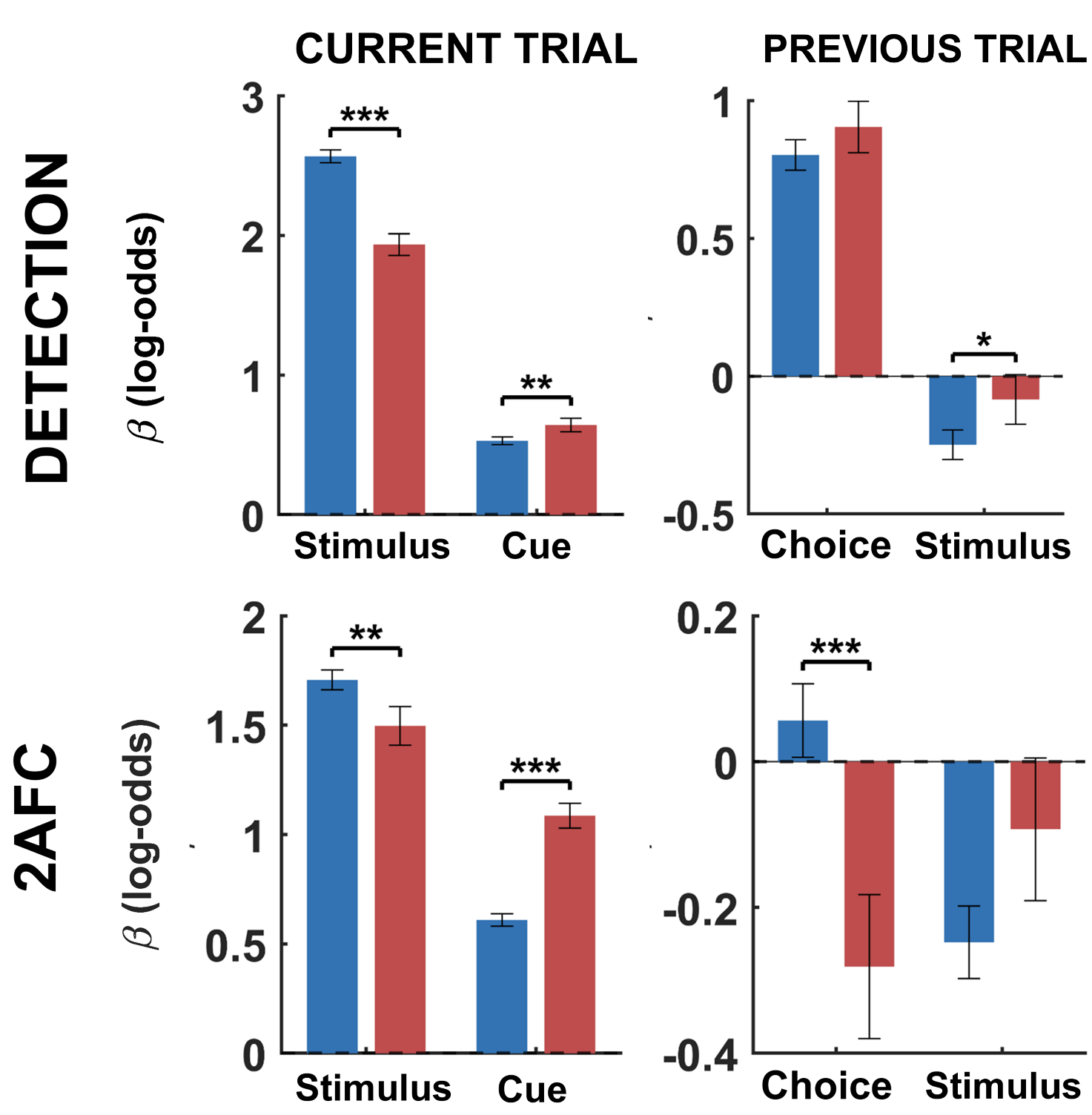


**Figure S3. Modulation of perceptual biases by psychosis-like traits in two perceptual decision-making tasks.** *Bar plots illustrate how High- versus Low-PLE participants (highest vs lowest terciles) differ in the estimated weighting of sensory evidence (Stimulus _t_), explicit cues (Cue _t_), and history terms (Choice _t-1_, Stimulus _t_ ₋₁) in the detection task (top) and the motion discrimination task (bottom). In detection, High-PLE participants show reduced sensory weighting (Stimulus _t_ × PLEgroup: β = −0.462, p < .001) and increased cue reliance (Cue _t_ × PLEgroup: β = 0.159, p < .001), with no significant modulation of choice repetition (Choice _t-1_ × PLEgroup: β = −0.098, p = 0.163). Stimulus-history repulsion is significantly attenuated (Stimulus _t_ ₋₁ × PLEgroup: β = 0.191, p = 0.0046). In motion discrimination, High-PLE participants again show reduced sensory weighting (Stimulus _t_ × PLEgroup: β = −0.193, p = 0.0031) and increased cue reliance (Cue _t_ × PLEgroup: β = 0.124, p = 0.0033), alongside reduced choice persistence / increased alternation (Choice _t-1_ × PLEgroup: β = −0.273, p < .001). The modulation of stimulus-history effects is not significant in this task (Stimulus _t_ ₋₁ × PLEgroup: β = 0.109, p = 0.131).*
